# Tamoxifen inhibits chemokinesis in equine neutrophils

**DOI:** 10.1186/s13620-018-0133-1

**Published:** 2018-10-23

**Authors:** Natalia Morales, Claudio Henriquez, Jose Sarmiento, Benjamin Uberti, Gabriel Moran

**Affiliations:** 1 Department of Pharmacology, Faculty of Veterinary Sciences, Universidad Austral de Chile, Valdivia, Chile; 2 Department of Physiology, Faculty of Medicine, Universidad Austral de Chile, Valdivia, Chile; 3 Department of Veterinary Clinical Sciences, Faculty of Veterinary Sciences, Universidad Austral de Chile, Valdivia, Chile

**Keywords:** Tamoxifen, Neutrophils, Chemokinesis, Horses

## Abstract

Neutrophils are terminally differentiated innate effector cells at the first line of host defense. Neutrophil migration within tissues is complex and involves several steps, during which these cells must be able to interpret a variety of chemical and physical signals. Exacerbated neutrophil activity can be harmful to surrounding tissues; this is important in a range of diseases, including equine asthma. Tamoxifen (TX) is a non-steroidal estrogen receptor modulator with effects on cell growth and survival. Previous studies showed that TX treatment in horses with induced acute pulmonary inflammation promoted early apoptosis of blood and bronchoalveolar lavage fluid (BALF) neutrophils, reduction of BALF neutrophil content, and improvement in animals’ clinical status. Further, TX dampens chemotactic index and respiratory burst production in vitro. The aim of this study was to provide information on the effect of TX on chemokinesis in peripheral blood neutrophils from five healthy horses. Results showed that neutrophils increased migration and travelled distance in response to IL-8; but in the presence of TX, IL-8 did not produce neutrophil migration. This suggests that TX has an inhibitory effect on the kinesis of equine peripheral blood neutrophils stimulated with IL-8. However, further studies are required to fully understand the signaling pathways of TX on neutrophil chemokinesis.

## Introduction

Neutrophils are terminally differentiated innate effector cells at the first line of host defense. They are capable of rapid deployment of a myriad of effector mechanisms to combat invading pathogens or clear damaged cells [1]. Because neutrophils rapidly migrate into inflammatory foci via diapedesis and chemotaxis, neutrophil recruitment has long been considered a hallmark of inflammation. Neutrophil chemotaxis is enhanced by several agents [2]. In addition to increased expression of adhesion molecules and receptors resulting from exocytosis, priming agents increase actin reorganization [3], and enhance chemokinesis and chemotaxis [4, 5]. Treatment of neutrophils with PAF, IL-8, or TNFα on their own, induces chemokinesis [6].

One of the diseases in which neutrophils play an important role in the equine airways is asthma, previously termed recurrent airway obstruction (RAO) [7–9]. Equine asthma is a chronic disease that develops in horses following stabling and exposure to dusty hay and straw [10]. The disease is characterized by pulmonary neutrophilia and excessive mucus production, resulting in reduced dynamic lung compliance and increased pulmonary resistance and pleural pressure excursions [11]. In asthma-affected horses, cytokine expression (including IL-8, IFN-gamma and TNF-alpha) is increased in bronchoalveolar lavage fluid (BALF) cells [10, 12, 13].

Tamoxifen (TX) is a synthetic non-steroidal anti-estrogen agent that is widely used for treating all stages of breast cancer and has been approved for the prevention of breast cancer in high-risk women [14, 15]. Our research group has previously shown that TX increases in vitro early apoptosis of granulocytic cells from horse peripheral blood and BALF [16]. Furthermore, Borlone et al. [16] showed that TX dampens chemotactic index and respiratory burst production in equine peripheral blood neutrophils in vitro. Our data also suggest that TX has the ability to induce apoptosis of granulocytic cells from peripheral blood and BALF obtained from horses with induced acute lung inflammation, with a concomitant improvement in their clinical status [17]. Given these findings, we hypothesized that TX inhibits the kinetic capacity of equine neutrophils in vitro*.* The aim of this study was to evaluate the effect of TX on chemokinesis in peripheral blood neutrophils from healthy horses.

## Material and methods

### Horses

Five clinically healthy adult horses ranging in age from 8 to 12 years, belonging and housed at Universidad Austral de Chile veterinary teaching hospital were enrolled in this study. There were four mares and one gelding respectively, of mixed breed, weighing 420–450 kg. All belonged to the University teaching herd for at least 3 years prior to the study, during which time they were systemically healthy. They were kept on pasture, and grass fed with free access to water. To ensure the animals’ health, qualified veterinarians performed physical examinations before sample collection for the duration of the study. All animals underwent complete blood cell counts prior to enrolment in the study, in order to exclude subclinical infections. All procedures were approved by the Universidad Austral de Chile Bioethics Committee for the Use of Animals in Biomedical Research (approval resolution n° 251/2016).

### Blood sampling and neutrophil isolation

The isolation of blood leukocytes was done as previously described by our group [16, 17]. Briefly, 10 mL of blood obtained by jugular venipuncture was placed in sterile tubes containing 1 mL of 3.8% *w*/*v* trisodium citrate. Blood was placed on a discontinuous density gradient (Percoll ® GE Healthcare), with 4 mL of 85% Percoll in the bottom of a 15 mL tube and 4 mL of 70% Percoll above. After centrifugation (45 min, 670 g), the upper layer contained mononuclear cells and the lower layer contained granulocytes. Both layers were aspirated for further processing. Cells were subsequently prepared for bioassays.

### Kinetic measurements of neutrophils

Kinetic measurements of neutrophil activity were evaluated using real-time microscopic visualization under constant flow of HBSS 1 mM Ca^2+^ (bath solution) as was previously described by our group [18]. 1 × 10^6^ cells ml-1 in HBSS 1 mM Ca^2+^ were seeded in clean coverslips without coating molecules for 20 min at 37 °C. Cells were placed into the thermal stage chamber (Brook Industries). Non-adherent cells were eliminated by the application of constant flow (1.5 ml/min) of bath solution using a peristaltic pump (model 7615–72 from Ismatec SA, Cole-parmer Instrument Company, IL, USA). After 10 min of basal recordings, cells were exposed to 15 ml of bath solution containing 0.1% DMSO; the cells were then exposed to 15 ml of bath solution with 30 nm IL-8 and 0.1% DMSO. Finally, the same cells were exposed to 15 ml of bath solution with 30 nm IL-8 and 10 μM tamoxifen. Stacks was collected with every 10 s using an AxioCam MRc5 (Carl Zeiss). For data analysis, total length of the cell path and average velocity were determined for 10–11 cells in the optical field using the Manual Tracking plugin of ImageJ. Sigma Plot (Systat Software Inc., version 11.0) was used for generation of polar plot graphs.

## Results and discussion

**Fig. 1:**
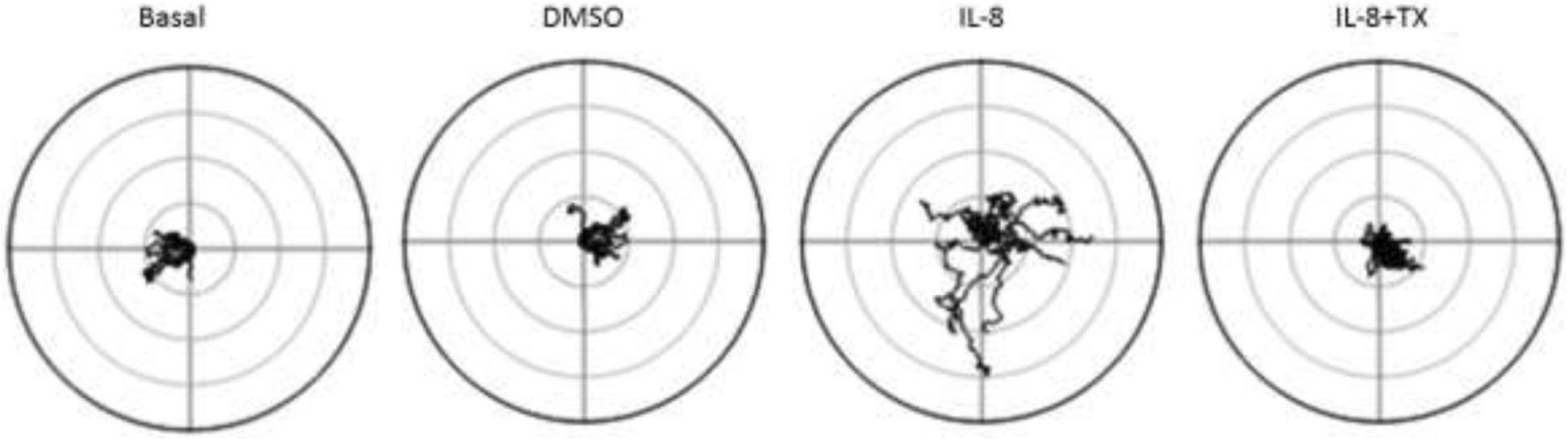
Polar plots of real-time neutrophil migratory capacity in different constant flow solutions. Basal: HBSS 1 mM Ca2+ (bath solution); DMSO: bath solution + 0.1% DMSO; IL-8: bath solution + 0.1% DMSO + IL-8 30 nm; IL-8 + TX: bath solution + IL-8 30 nm + tamoxifen 10 μM

To further explore the role of TX in the neutrophil migratory process, we studied chemokinesis using video microscopy. On this occasion, we chose to use peripheral blood neutrophils from healthy horses because these conditions provide neutrophils in an inactivated state, required for this functional test. Moreover, blood samples are much easier to obtain than BALF samples, and neutrophil counts in BALF from healthy horses are very low. Figure 1 shows the trajectory of cells under the different conditions tested. Neutrophils showed no migratory capacity either in their basal state or with added DMSO flow (TX vehicle). Neutrophil migration and travelled distance increased in response to IL-8; but in the presence of TX, IL-8 was unable to stimulate neutrophil migration, producing comparable readings to the basal and DMSO groups. Neutrophil migration within tissues is complex and involves several steps, during which these cells must be able to interpret a variety of chemical and physical signals. Moreover, cell migration is largely dependent on the polarization of several major proteins in the plasma membrane including ion channels [19]. Furthermore, during neutrophil chemotaxis, various cytoskeletal arrangements cooperate to optimize migration in response to chemoattractants [20]. Our results show that TX has the ability to decrease kinesis in neutrophils stimulated with IL-8. However, the signaling pathway by which TX exerts its inhibitory effect of neutrophil kinesis is not yet elucidated. One possibility to explore is the effect of TX on ion channels or on changes associated with cytoskeletal-associated proteins.

In asthma-affected horses, neutrophils migrate within hours into the airway lumen, followed by the development of airway obstruction and a late phase of migration [21, 22]. A Type III hypersensitivity reaction explains, in part, the neutrophilic inflammation in these patients’ airways, but the factors initiating neutrophilia have not been completely elucidated [23]. However, IL-8 plays an important role in airway inflammation in equine asthma. This study shows that stimulation of cells with IL-8 (30 nM) produces a significant increase in the movement of polymorphonuclear neutrophils. An increase in the concentration of IL-8 in BALF has been demonstrated in asthma-affected horses after antigenic challenge [12, 13, 21]. Other authors reported upregulated IL-8 mRNA expression in BALF cells and endobronchial biopsies from asthma-affected horses in acute crisis [21, 24]. Some authors also suggest that alveolar macrophages can contribute to airway inflammation through the release of IL-8, macrophage inflammatory protein-2 (MIP-2) and TNF-α [12, 13, 25]. Our data from this first report suggests that TX has an inhibitory effect on the kinesis of equine peripheral blood neutrophils stimulated with IL-8.

## Conclusion

Previous studies show that TX increases in vitro early apoptosis of granulocytic cells from horse peripheral blood and BALF [26]. TX also dampens chemotactic index and respiratory burst production in equine peripheral blood neutrophils [16]; and this study shows that TX inhibits the chemokinetic effects of IL-8 in equine neutrophils. Furthermore, recent results show that incubation of neutrophils with 5 μm of tamoxifen induces their efferocytosis by macrophages [27]. All of the above could partially explain the anti-inflammatory effect of TX in horses with airway inflammation described by our group [17]. However, more in vivo and in vitro studies are required to fully understand the mechanisms of action of TX on neutrophils, in order to elucidate by which mechanism TX produces clinical improvement in equine airway inflammation.

## Abbreviation

BALF: Bronchoalveolar lavage fluid
DMSO: Dimethylsulfoxide
RAO: Recurrent airway obstruction
TX: Tamoxifen
